# DNA damage and repair dependencies of ionising radiation modalities

**DOI:** 10.1042/BSR20222586

**Published:** 2023-10-03

**Authors:** Emma Melia, Jason L. Parsons

**Affiliations:** Institute of Cancer and Genomic Sciences, University of Birmingham, Edgbaston, Birmingham B15 2TT, U.K.

**Keywords:** complex DNA damage, DNA repair, ionising radiation, linear energy transfer, radiotherapy, relative biological effectiveness

## Abstract

Radiotherapy is utilised in the treatment of ∼50% of all human cancers, which predominantly employs photon radiation. However, particle radiotherapy elicits significant benefits over conventional photons due to more precise dose deposition and increased linear energy transfer (LET) that generates an enhanced therapeutic response. Specifically, proton beam therapy (PBT) and carbon ion radiotherapy (CIRT) are characterised by a Bragg peak, which generates a low entrance radiation dose, with the majority of the energy deposition being defined within a small region which can be specifically targeted to the tumour, followed by a low exit dose. PBT is deemed relatively low-LET whereas CIRT is more densely ionising and therefore high LET. Despite the radiotherapy type, tumour cell killing relies heavily on the introduction of DNA damage that overwhelms the repair capacity of the tumour cells. It is known that DNA damage complexity increases with LET that leads to enhanced biological effectiveness, although the specific DNA repair pathways that are activated following the different radiation sources is unclear. This knowledge is required to determine whether specific proteins and enzymes within these pathways can be targeted to further increase the efficacy of the radiation. In this review, we provide an overview of the different radiation modalities and the DNA repair pathways that are responsive to these. We also provide up-to-date knowledge of studies examining the impact of LET and DNA damage complexity on DNA repair pathway choice, followed by evidence on how enzymes within these pathways could potentially be therapeutically exploited to further increase tumour radiosensitivity, and therefore radiotherapy efficacy.

## Introduction

Conventional radiotherapy, utilising X-rays (photons), is the most common line of treatment for human malignancies, ever since its first application in treating a patient with breast cancer in 1896 [1]. To date, approximately 50% of patients receive some form of radiotherapy, either as a monotherapy or in combination with surgery or chemotherapy [2]. However, due to the continual release of energy along the radiation track of photons, and therefore high entrance and exit doses, this can deliver an unnecessary radiation dose to the surrounding healthy tissues and organs at risk, leading to acute and long-term adverse effects. In the last several decades, there has been the emergence of more precision-targeted radiotherapy techniques, specifically using particle ions, such as protons and carbon ions. Particle beam therapy benefits from a significantly lower entrance and exit dose, and the delivery of radiation more precisely to the tumour via the Bragg peak. Proton therapy was made commercially available in the 1970s, and this was followed by the completion of the first heavy ion medical accelerator in Japan in 1993, with clinical trials being conducted on human malignancies a year later [3]. The beam characteristics of particle therapy are not only favourable over photons through defined dose deposition, but also due to the increases in linear energy transfer (LET) and associated relative biological effectiveness (RBE) at and around the Bragg peak. LET is defined as the spatial energy deposition along the radiation track, resulting in the ionisation of key cellular molecules, particularly DNA. Photons are sparsely ionising and therefore considered to be low-LET, whilst carbon ions are densely ionising and consequently high-LET. Protons are considered relatively low-LET, although this can vary across the radiation track and is highest at the distal fall-off of the Bragg peak. RBE refers to the dose required to achieve equivalent biological effects between different radiation modalities, with photons used largely as a reference. The higher the RBE the greater the tumour cell killing, and the RBE is known to increase with increasing LET of the radiation type [4]. In addition to particle beam therapy, there has been more recent advances in the development and application of boron neutron capture therapy (BNCT). BNCT involves treatment with a boronated compound and irradiation with low energy thermal neutron beams, which creates high-LET α-particles that introduces extensive DNA damage in a more localised region [5]. Excitingly, in relation to healthy tissue sparing, there has been some very recent progressive developments in the utilisation of ultra-high dose rate (FLASH) radiotherapy. FLASH can be combined with particle ions, such as protons, to maximise both tumour killing and healthy tissue sparing [6].

Irrespective of the radiation type or quality, the effectiveness of radiotherapy relies heavily on the introduction of DNA damage to the tumour cells to induce cell death. The greater the LET of the radiation, the increased clustering and complexity of the DNA damage, which translates to increased cell killing. However, cells have developed highly specialised DNA damage response mechanisms allowing for fast detection and ultimately repair of the damage. The aim of radiotherapy is to introduce sufficient DNA damage into the cancer cells that overwhelms their capacity for repair leading to cell death. However, often tumours are inherently radioresistant, due to factors such as accelerated DNA damage signalling or repair mechanisms. Tumours are also hypoxic (lacking oxygen) which creates a significant barrier to effective treatment, particularly using conventional photon radiotherapy. Therefore, these biological radioresistance mechanisms require a more detailed understanding in order for these to be exploited therapeutically to increase the efficacy of radiotherapy treatment, but also to fully explore alternative radiotherapy modalities (e.g. protons, FLASH and BNCT) for optimal patient treatments.

In this review, we will discuss the different types of ionising radiation (IR), specifically their physical and biological properties, as well as highlighting the specific DNA damage and repair pathways that are responsive to these different radiation modalities with emphasis on the relationship to LET. Finally, we will highlight evidence regarding combinatorial treatments, particularly targeting the cellular DNA damage response, in an attempt to increase sensitisation of tumour cells with radiation of differing LET.

## Radiation modalities

### X-rays

**Figure 1:**
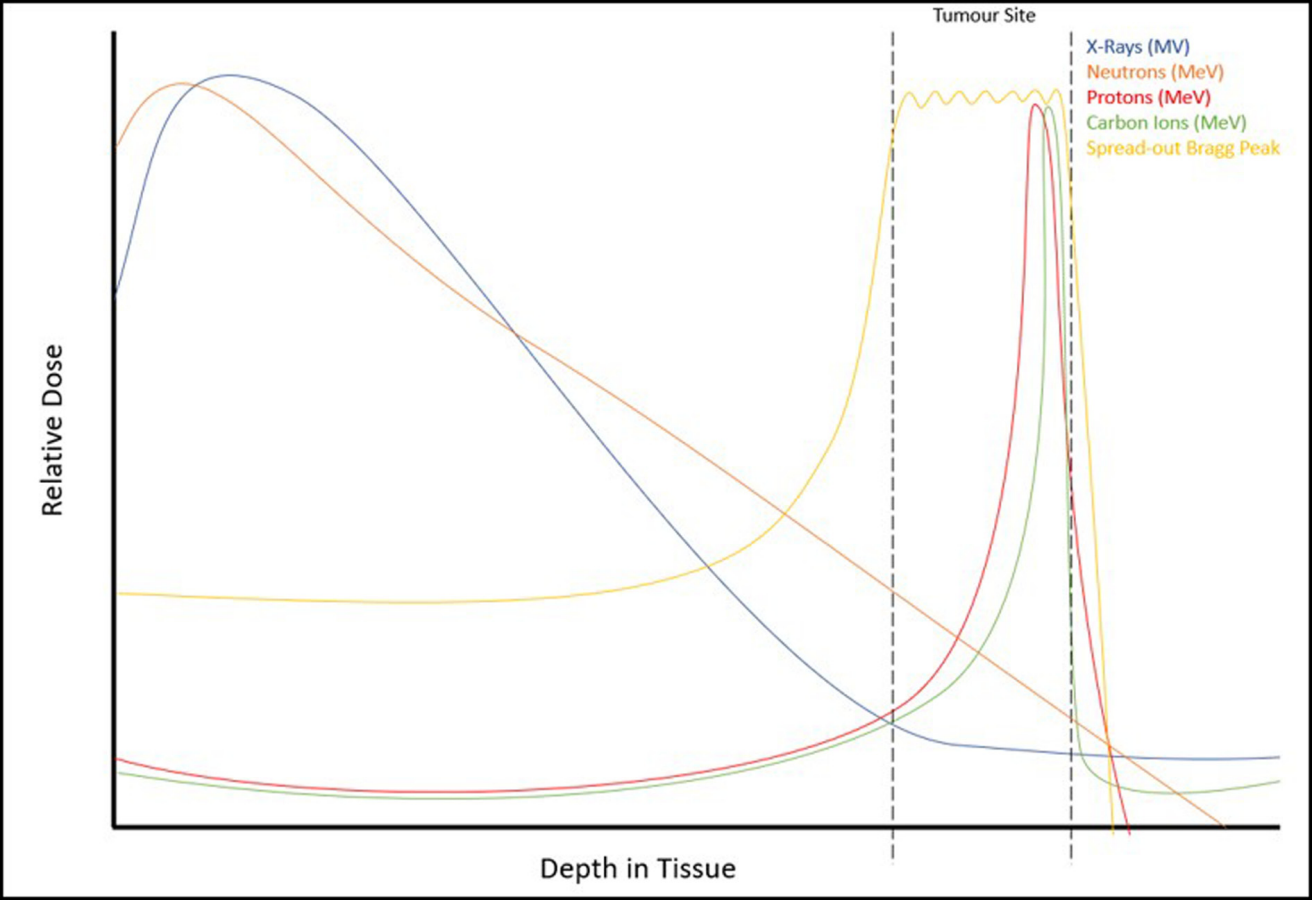
Relative dose curves for the different types of radiation X-rays (MV, blue line) have a high entrance dose, with a relatively lower dose being applied to the tumour, similar to that of neutrons (MeV, orange line). Protons (MeV, red line) have a low entrance and exit dose, depositing the majority of their energy in the Bragg peak which can be targeted at the tumour, as the dose curve progresses and the LET of the protons increases. Carbon ions (MeV, green line) have a very high LET, and similar to protons they display low entrance dose levels. However, the Bragg peak with carbon ions is much steeper and has a quicker distal fall off compared with protons. There is also a post-Bragg peak tail region where the dose slightly increases outside the tumour range. A pristine Bragg peak can be manipulated by using multiple beams of varying initial energy to target the whole tumour depth, which yields a spread-out Bragg peak (yellow line).

The current most common form of radiation treatment of cancer worldwide is in the form of photons (X-rays). Photons have no mass or charge and are capable of causing ionising damage to tissues and cells either directly or, most commonly, indirectly through the generation of reactive oxygen species (ROS). X-rays are a low-LET radiation source where the track structure is sparsely ionising, given rise to largely isolated damage to critical cellular structures, such as DNA. Photon radiotherapy has many side effects as it is known to deposit the majority of its energy immediately upon entry into the tissue, and continuously along its radiation track (Figure 1). Consequently, healthy tissues surrounding the target tumour receive significantly more radiation dose resulting in potentially acute or severe adverse side effects, whereas the tumour site subsequently receives a much lower X-ray dose. Despite this, photon radiotherapy is still being utilised in ∼50% of the treatment of all human cancers [2].

### Protons

The use of protons in cancer therapy was first suggested by Robert R. Wilson in 1946, following investigations into controlling the proton beam depth by manipulating proton energy [7]. Proton beam therapy (PBT) utilises single beams of excited protons to emit doses of radiation to the tumour. PBT is favourable over conventional photon irradiation mainly due to its physical properties. Here, PBT displays a low entrance dose which minimises the damage to normal tissues, but as the protons slow down and come to rest at the target tumour site, the majority of the energy is deposited in a small finite region known as the Bragg peak (Figure 1). This is followed by a low exit dose which again minimises the damage to the associated normal tissues and organs at risk in proximity to the tumour being treated. Protons are positively charged particles and are deemed low-LET, although throughout the Bragg peak there are increases in LET that are above those achieved with photon radiotherapy, but not as high as other particle irradiations, such as carbon ions. This characteristic of PBT results in a variable LET across the radiation track, and which can contribute to some degree of biological and clinical uncertainty. The depth at which protons reach in the body can be easily manipulated by controlling their initial energy, resulting in high precision in the targeting of the tumour site. In the clinical use of PBT, the dose is measured relative to the dose of conventional photon therapy with an equivalent relative biological effect (RBE). Due to the increases in LET, an RBE of PBT of 1.1 is widely used, essentially assuming that PBT has a 10% higher biological effectiveness compared with the equivalent dose of photons [8]. However, this is highly controversial and there is an indication that PBT should have a variable RBE across the Bragg peak due to the changes in LET [9]. The aforementioned characteristics of PBT can be manipulated in a clinical setting, in order for the whole tumour to be targeted taking into consideration the tumour size and depth. This involves applying multiple protons beams with varying initial energy to create a spread-out Bragg peak (SOBP) (Figure 1), which results in a similar energy deposition across the tumour site [10].

### Carbon ions

Carbon ions have an extremely large mass and charge, compared with other particle therapies, such as PBT, resulting in a more precise beam-line path. Carbon ion radiotherapy (CIRT) utilises single beams of energised carbon ions that are characterised by a more direct beam path and a sharper Bragg peak compared with protons (Figure 1) [11]. Additionally, the RBE of carbon ions has been shown to be 2- to 3-fold higher than that of PBT, greatly owed to a substantially higher LET [12]. This increased biological effectiveness of CIRT is as a consequence of the increased complexity of damage induced, due to the dense radiation track structure. Similar to PBT, by controlling the initial energy of the carbon ions, a SOBP can be used to target the whole tumour depth. In addition to the direct damage to tumour cells via carbon ion introduction at the Bragg peak, there is also the production of secondary particles that can surpass the Bragg peak, creating a tail and inducing catastrophic damage beyond the target tumour region and potentially into the healthy tissues [13]. Despite this, CIRT is more favourable than photon radiotherapy or PBT with regards to inducing cancer cell killing due to the significantly higher LET, but there are also significant radiobiological benefits as CIRT is less sensitive to oxygen concentrations (hypoxia), inherent radiosensitivity of the tumour cells as well as cell cycle distribution.

## Boron neutron capture therapy

Boron neutron capture therapy (BNCT) was first pioneered as a potential therapeutic in oncology by G.L Locher, in the 1930s [14]. The basic principles of BNCT involves the accumulation of stable boron-10 (^10^B) isotopes in cancer cells, predominantly through the amino acid transporter LAT-1 that is upregulated in tumour cells [15]. Following this, the cancer cells are subjected to neutron beam irradiation, which consists of low energy neutrons (also referred to as ‘thermal’ neutrons), which cause the generation of unstable ^11^B isotopes, which then undergo a nuclear reaction to generate high LET helium (^4^He) and lithium (^7^Li) ions [16]. It is the generation of these highly ionising particles which results in the significant amount of cellular and molecular (DNA) damage. As a result of the preferential accumulation of ^10^B in cancer cells, this damage is very dense and highly localised, providing maximal healthy tissue sparing. However, low energy neutrons can also be captured by naturally occurring protons and nitrogen (^14^N) ions in the cell, resulting in the production of low-LET γ-rays and high-LET carbon ions. This capture can potentially pose an adverse risk to healthy tissues in the neutron beam path.

## FLASH

More recently, there has been a growing interest in increasing the delivery rate of radiotherapy, particularly at ultra-high dose rates (FLASH), driven in 2014 by the Vozenin Group [17]. This and the immediate following studies were performed using linear electron accelerators, however FLASH can be delivered with any radiation modality, such as PBT [6] and CIRT, where the dose rates can be manipulated to exceed 40 Gy/sec. This dose rate is considered the threshold for observing the ‘FLASH effect’. The principle behind the ‘FLASH effect’ is the significant normal tissue sparing that is observed, whilst the radiation still displays its impact on the tumour in terms of cell killing and prevention of tumour growth [17,18]. FLASH radiotherapy therefore can allow for an increase in radiation dose to be delivered to the tumour, which is important for inherently radioresistant tumours, with no apparent increased adverse side effects due to minimal impact on the surrounding normal tissues and organs at risk. Despite the significant clinical potential for FLASH radiotherapy, there are a number of unanswered questions relating to the biological mechanisms contributing to the ‘FLASH effect’, including the role of oxygen, DNA damage, metabolic and immune cell effects, in addition to whether the radiobiology of high-LET radiation is maintained at ultra-high dose rates.

## DNA damage and repair

The effectiveness of radiotherapy using IR relies heavily on the introduction of sufficient DNA damage to cancerous cells, ultimately leading to cell death. As mentioned above, this damage can occur due to direct interaction with the DNA, but in the case of low-LET radiation, this occurs largely through an indirect mechanism involving the radiolysis of water. This process creates ROS that when produced in close proximity (<10 nm) to the DNA causes damage [19]. There are many different types of damage that can result from exposure of the DNA to radiation, but these can be broadly separated into DNA base lesions, DNA single strand breaks (SSBs) and DNA double strand breaks (DSBs). The relative numbers of these DNA lesions generated by low-LET radiation demonstrate an increased frequency of DNA base damages and SSBs (∼1,000 each/Gy) compared with DNA DSBs (∼40/Gy). However, a signature of IR is the ability for this to generate clustered/complex DNA damage (CDD), containing two or more DNA lesions within one or two helical turns of the DNA [20]. The simplest form of a CDD site is a DSB, although CDD can actually be composed of a variety of different DNA lesions, making this challenging to accurately measure both *in vitro* and *in vivo*. It has been suggested using mathematical models that up to 50% of DSBs can have an associated strand break or base damage in close proximity even following low-LET radiation [21,22]. Importantly, the frequency and complexity of CDD increases with increasing LET, such that with high-LET α-particles the majority (>90%) of DSBs induced are complex [23].

**Figure 2:**
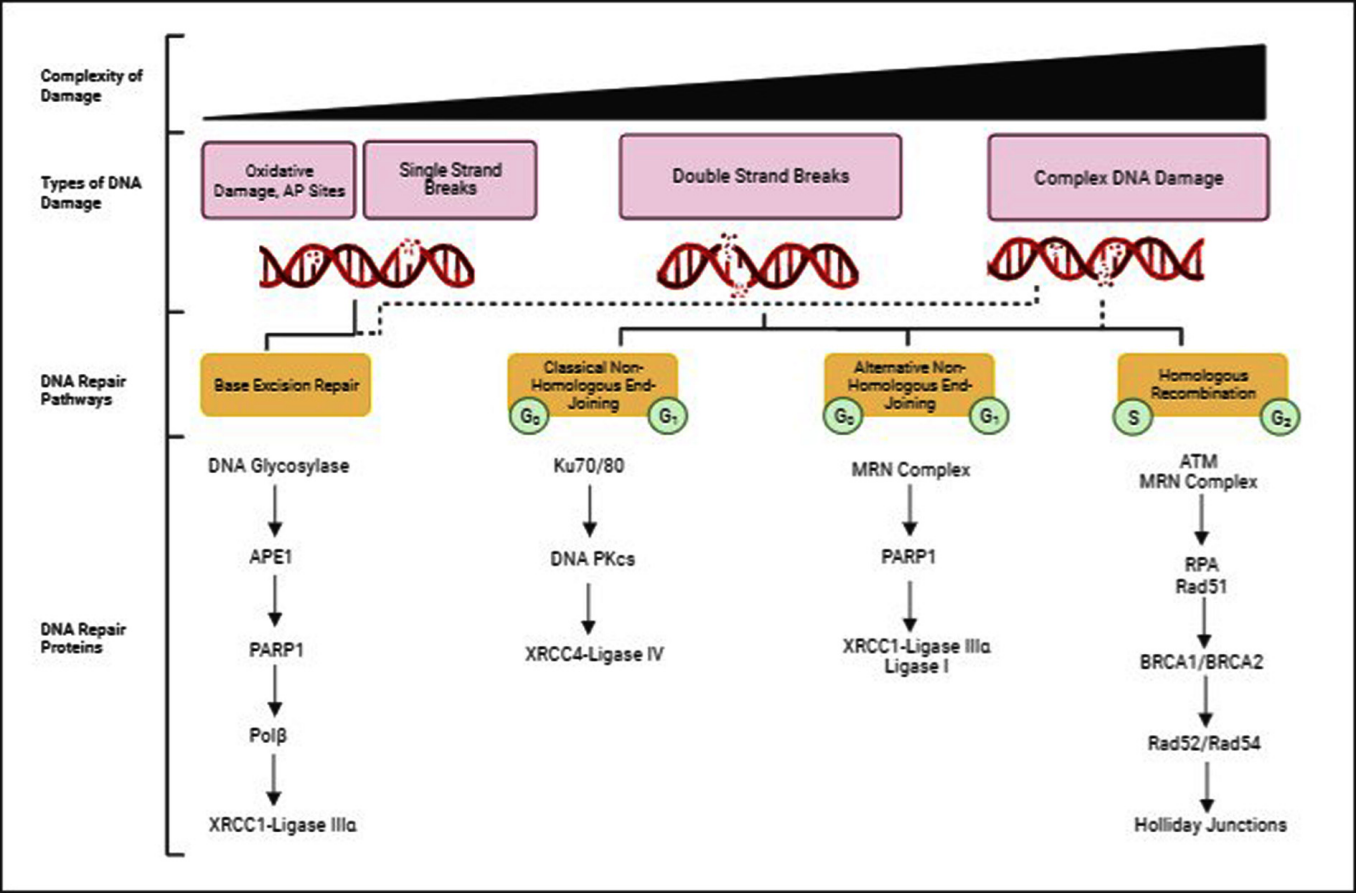
Simplified overview of IR-induced DNA damage and repair pathways IR induces DNA damage of varying complexity, ranging from oxidative damage, AP sites and SSBs, to DSBs and CDD. Oxidative damage, AP sites and SSBs are repaired by the BER pathway, that is constitutively active. BER involves recognition of the DNA base damage by damage-specific DNA glycosylases to create an AP site, which is then incised by APE1 to create a SSB with 5′-dRP and 3′-hydroxyl ends. The SSB is bound by PARP1 that recruits Pol β to remove the dRP moiety and simultaneous inserts a new, undamaged nucleotide into the gap. DNA ligation is then performed by XRCC1-Lig IIIα complex. DSBs can be repaired by NHEJ (both classical and alternative pathways), mainly in G_0_ and G_1_ cell cycle phases, or by HR which is active in only S and G_2_ phases. C-NHEJ involves Ku70/80 binding to the exposed DNA ends, which recruits DNA-PKcs and XRCC4-Lig IV to allow for DNA ligation. A-NHEJ involves DSB recognition via the MRN complex that resect the DNA ends, this enables PARP1 binding and subsequently DNA ligation is performed by either XRCC1-Lig IIIα or Lig I. HR involves recognition of the DSB by ATM and the MRN Complex, which stimulates DNA end resection, allowing for RPA to coat the single stranded DNA formed. RPA recruits and activates Rad51, whose binding is accompanied by BRCA1. The sister chromatid is then invaded through a BRCA2-dependent processes, which allows for homologous DNA synthesis through Rad52/Rad54. Following this, there is the formation and resolution of Holliday junctions to complete the DSB repair process. Given the nature of CDD, which can consist of many different types of DNA lesions, this is likely to use a combination of the different BER and DSB repair pathways to resolve the damage (indicated by the dotted lines).

DNA base damage and SSBs are all repaired by proteins of the base excision repair (BER) pathway (Figure 2) [24,25]. In this pathway, the base damage is recognised and excised by a damage-specific DNA glycosylase, of which 8-oxoguanine DNA glycosylase (OGG1) and endonuclease III homologue (NTH1) are the main enzymes responsible for the repair of IR-induced oxidative DNA base damage. This then stimulates AP-endonuclease-1 (APE-1) to bind to the subsequent AP site formed and to create an incision in the DNA backbone leaving a SSB flanked by 5′-deoxyribose phosphate (dRP) and 3′-hydroxyl ends. Poly(ADP-ribose) polymerase-1 (PARP1) binds to the SSB with a high affinity and as well as protecting the DNA ends, recruits a number of enzymes, including DNA polymerase β (Pol β). Pol β is then able to remove the dRP moiety and fill the single-nucleotide gap, before DNA ligation occurs due to the action of the complex of X-ray cross-complementing protein 1 and DNA ligase IIIα (XRCC1-Lig IIIα). Although isolated DNA base damage and SSB lesions are produced in abundance following IR, the time and difficulty to repair these is relatively low. However, IR is capable of creating more complex damage, including DSBs and CDD that present more of a challenge to the DNA repair machinery and are deemed the major lesions contributing to IR-induced cell lethality.

DSBs consist of breaks in both of the opposing phosphodiester bonds of the DNA backbone and are most commonly repaired by the error-prone non-homologous end-joining (NHEJ) pathway, which is active throughout the cell cycle. NHEJ can be further sub-divided into classical and alternative pathways (Figure 2). Classical NHEJ (c-NHEJ) involves Ku70/80 complex binding to the DSB ends, resulting in DNA-dependent protein kinase, catalytic subunit (DNA-PKcs) binding, and activation of a variety of limited end-processing, involving enzymes such as Artemis and polynucleotide kinase phosphatase (PNKP) [26]. Following the processing of the DSB, DNA ligation occurs through the action of the complex of X-ray cross-complementing protein 4 and DNA ligase IV (XRCC4-Lig IV). Alternative NHEJ (a-NHEJ) involves DSB detection by the MRE11-Rad50-NBS1 (MRN) complex that performs a limited amount of DNA end resection and allows PARP1 binding to the DNA ends. DNA ligation is then performed by either XRCC1-Lig IIIα or Lig I [27]. DSBs can be simple, ‘clean’ breaks, or the resultant DNA ends can be more complex, which determines the amount of end processing necessary during the repair process [28]. In contrast with NHEJ, homologous recombination (HR) is error free however it is only functional when the sister chromatid is exposed in S and G_2_ phases of the cell cycle [29]. HR involves DSB detection by the ataxia telangiectasia mutated (ATM) protein kinase and the MRN complex that causes resection of the DNA ends. The resulting and exposed single stranded DNA ends that are formed are coated with replication protein A (RPA), which then recruits ataxia telangiectasia and Rad3 related interacting protein (ATRIP), and subsequently ataxia telangiectasia and Rad3 related (ATR) or Rad51. In the case of ATR activation, checkpoint kinase 1 (CHK1) is then activated and ultimately induces cell cycle arrest at the G_2_/M checkpoint, to allow for DNA repair via the HR pathway. The binding of Rad51 then recruits and activates breast cancer genes 1 and 2 (BRCA1 and BRCA2), which allows for BRCA2-mediated invasion of the sister chromatid that acts as a template for repair. DNA synthesis is then assisted by Rad52 and Rad54 which ultimately results in the formation and resolution of Holliday junctions (Figure 2). In terms of IR with a higher LET, there will be an increased amount and complexity of CDD being formed. CDD is unsurprisingly complex in nature and can generally be categorised as complex DSBs or non-DSB clusters [30]. Consequently, it is likely that DSB-associated CDD has more of a reliance on the DSB repair pathways, compared with the requirement for the BER pathway during the resolution of non-DSB CDD. However, it is likely that due to the different types of DNA lesions that constitute a CDD site, particularly with high LET radiation such as CIRT, then CDD repair responses are likely to utilise a mixture of BER, NHEJ and HR pathways to resolve the damage (Figure 2).

## LET-dependent DNA damage and repair

Although it is widely accepted that there is an increase in the amount and complexity of CDD with increasing LET, surprisingly it is not yet clear of the involvement of the different DNA repair pathways relative to LET [31]. We have summarised some of the cell-based evidence examining the response to different radiation types and qualities below (Table 1). Following low-LET photon radiation, the majority of DNA damage is isolated base damage and SSBs that are often easily repaired and are mainly targets for the BER pathway [20]. Proton irradiation, although of relatively low-LET versus other forms of particle irradiation, compared with photon irradiation protons have increased LET at and around the Bragg peak. Here there is an increased generation of CDD, specifically in the distal end of the Bragg peak where the LET is greatest [31]. It has been debated for some time that the repair of DSBs following proton irradiation relies heavily on either NHEJ or HR, but the impact of LET also needs to be considered. A study was conducted in Chinese hamster ovary (CHO) and lung fibroblast cell lines deficient for DNA-PKcs or XRCC4 (essential for NHEJ) or XRCC2 and XRCC3 (Rad51 paralogs, essential for HR) [32] and irradiated with protons (200 MeV; 2.2 keV/µm) in the middle of the SOBP. The study determined via clonogenic survival assays that those cells deficient for NHEJ, specifically DNA-PKcs, were significantly more sensitive to proton irradiation, compared with their wild-type and HR deficient counterparts. Similar conclusions were also drawn from another study using CHO cells deficient for DNA-PKcs where cells were irradiated with protons (20 MeV) positioned in the entrance plateau (3.4 keV/µm) or in the Bragg peak (14 keV/µm) [33]. Here, it was observed that there was increase in induction, size and spatial distribution of γH2AX foci following proton irradiation, compared with γ-ray irradiation. These DSBs were seen to persist in cells deficient for DNA-PKcs following proton irradiation, however, surprisingly there were no differences observed with the varying LET of the protons. In our recent study, we also demonstrated that head and neck squamous cell carcinoma cells treated with inhibitors against DNA-PKcs (KU-57788) or ATM (KU-55933) showed greater radiosensitivity following proton irradiation compared with those treated with an ATR (VE-821) inhibitor, suggesting an important role for NHEJ in the repair of proton-induced DSBs [34]. However, cells were exposed to protons at the entrance plateau (60 MeV, 1 keV/µm), and so LET dependence towards the Bragg peak was not investigated. In contrast with the above, other reports have shown an increased reliance on HR following proton irradiation. Firstly, a study was performed in A549 and glioblastoma cells, with exposure to protons (138MeV) in the middle of the Bragg peak, and treatment with DNA-PKcs inhibitor (NU7026) or small interfering RNA (siRNA) for DNA-PKcs/Rad51. The study revealed via clonogenic survival assays that the mammalian cancer cells were much more radiosensitive to proton irradiation following depletion of Rad51 compared with a lack or inhibition of DNA-PKcs [35]. There was also an increase in the persistence of DSBs visualised by γH2AX foci following protons with Rad51 siRNA, therefore suggesting a reliance on the HR pathway for repair of proton induced-DSBs. Similar data were observed in CHO cells where HR-deficient cells or those treated with Rad51 siRNA were hypersensitive to proton irradiation compared with NHEJ-deficient cells [36]. These conclusions are further supported by a study using genetically modified mouse embryonic fibroblasts following exposure to protons (230 MeV) at both the entrance plateau and the mid-SOBP [37]. The results showed that Rad54 knockout cell lines were significantly more sensitive to proton irradiation, compared with wild-type and Lig IV knockout cell lines, but which was not LET-dependent. There was no significant decrease in the survival of double knockout cell lines (Rad54 and ligase IV), compared with Rad54 knockout alone, suggesting that the DNA damage response following proton irradiation was primarily reliant on HR. However surprisingly, Lig IV deficiency also resulted in a stronger sensitisation to protons versus photons. It was proposed through DNA repair analysis that Rad54 knockout cells showed a more pronounced delay in the repair of DSBs with SOBP protons versus photons. Additionally, using glioblastoma cell lines it was shown that DNA-PKcs deficiency had no impact on the comparative sensitisation to photons versus protons, whereas BRCA2 mutant and Capan-1 cells were hypersensitive to entrance and SOBP protons associated with delays in DSB repair. These results indicate a requirement for HR in response to proton irradiation, irrespective of LET. Nevertheless, and along with this conflicting data, it is difficult to compare the conclusions drawn from these studies due to differences in beam characteristics and/or cellular models, emphasising the need for a more systematic and standardised approach when experiments are performed.

**Table 1:** Dependency on specific DNA repair pathways following different types of IR

Radiation type	Beam characteristics	DNA repair protein	Cell type	Dominant repair pathway	Ref.
**PBT**	200 MeV, 2.2keV/µm, Mid-SOBP	DNA PKcs or XRCC4 knockout	AA8, V79	NHEJ	[32]
	20 MeV, 3.4 keV/µm Entrance plateau, 14 keV/µm Bragg peak	DNA PKcs deficient cells	CHO10B2	NHEJ	[33]
	60 MeV, 1 keV/µm, Entrance plateau	DNA PKcs Inhibitor	UMSCC-6, 74, 47, FaDu, A253, UPCI-SCC90	NHEJ	[34]
	138 MeV, Mid-SOBP	DNA PKcs inhibitor DNAPKcs/Rad51 siRNA	A549, M059K, M059J	HR	[35]
	138 MeV Mid-SOBP	XRCC3 knockout	AA8, CHO9, CHO-Irs1sf, CHO-XR-C1	HR	[36]
	230 MeV, Entrance plateau/Mid-SOBP	Rad54 and Ligase IV knockout	MEF, M059K, M059J, Capan-1, BxPC3	HR	[37]
**CIRT**	290 MeV, 50 keV/µm, Mid-SOBP	DNA PKcs or XRCC4, and XRCC2 or XRCC3 knockout	AA8, V79	NHEJ and HR	[32]
	290 MeV, 70 keV/µm	Ku70/80 or ligase IV knockout	CHO-xrs6, HFL-III	NHEJ	[38]
	290 MeV/18.3 MeV, 13, 50, 70 keV/µm/108 keV/µm, Mid-SPBO	Ligase IV or Rad54 knockout	MEF	NHEJ	[39]
	290 MeV, 70 keV/µm	Phosphorylation of CtIP and RPA	U2OS, HeLa, U251 and MEF	HR or a-NHEJ	[40]
**BNCT**		Activation of BRCA1/Rad51, not Ku70/80	Huh7	HR	[41]
		Activation of Rad51, Rad54, Ku70/80	WRO, Mel J	HR	[42]
		Ku70/80 knockout cells	CHO-K-1	NHEJ	[43]
		Ligase IV knockout	MEF	NHEJ	[44]
**Others**					
Iron ions	500 MeV, 200 keV/µm	Phosphorylation of CtIP and RPA	U2OS, HeLa, U251 and MEF	HR or a-NHEJ	[40]
Iron ions	1 GeV, 151 keV/µm	Rad51 siRNA	AA8, HCA2-hTERT, U2OS, TK6, WTK1	HR	[45]
Heavy ions	11.4 MeV/100 MeV (CIRT), Ranging 170-15,000 KeV/µm/90 keV/µm	RPA foci, activation of ATR, MRE11 and CtIP	U2OS, NFFhTERT	HR or a-NHEJ	[46]
α-particles	115 keV/µm	Analysis of 53BP1 and RPA foci	U2OS	HR	[47]
α-particles		Effects of ATM and DNA PKcs inhibitor	H460, 22rv1, HCT116, A549, H1299, MRC-5, HEK293	HR	[48]

In comparison with photons and protons, carbon ions have significantly increased LET resulting in highly CDD being formed that has substantially reduced repairability. Interestingly, whilst it is thought that there is more of a reliance on HR following exposure to protons with slightly higher LET, there is actually increasing evidence for the importance of NHEJ following CIRT. Utilising Ku80-deficient CHO cell lines and Lig IV-deficient human fibroblasts, both of which are unable to perform NHEJ, a significant increase in sensitivity to high-LET CIRT (290 MeV, 70 keV/µm) was observed compared with wild-type cells [38]. There was an associated delay in the autophosphorylation of DNA-PKcs, which persisted at higher levels post-irradiation when compared with low-LET photon irradiation conditions, particularly in Lig IV-deficient cells. These results indicate that the presence of CDD-induced by CIRT delays the activation of the NHEJ pathway, which is essential for repair. These results were exacerbated when cells were exposed to iron ions, with a much higher LET (500 MeV, 200 keV/µm), highlighting the importance of the LET on the DNA damage induced and the consequential repair pathway activated. This evidence was further supported by a study performed on mouse embryonic fibroblasts deficient for either Lig IV or Rad54 [39]. This revealed that impairing both NHEJ and HR repair pathways had the most profound effect on radiosensitisation to CIRT (290 MeV, 70 keV/µm), although the response was very similar to Lig IV deficient cells, indicating that high-LET-induced DNA damage primarily relies on NHEJ for repair. Similar conclusions were drawn from a study utilising CHO cell lines demonstrating a significant enhancement of radiosensitivity of NHEJ-deficient cells following CIRT (290 MeV, 50 keV/µm), highlighting the importance of the repair pathway under these conditions [32]. However, it was also shown that HR-deficient cells had a greater radiosensitivity following CIRT compared with protons and photons emphasising the increasing importance of HR following higher LET radiation. In line with this observation, a study in human cancer cells (U2OS and HeLa) and mouse embryonic fibroblasts revealed that the complexity of the damage following CIRT (290 MeV, 70 keV/µm) resulted in an increase in DNA end resection, as analysed through CtIP and RPA phosphorylation, indicative of the early phase of the HR pathway prior to DNA repair [40]. Similar results were also seen in response to high-LET iron ions, which therefore suggests a dependence on HR for the repair of CDD generated by higher LET radiation.

BNCT is known to generate high LET helium and lithium ions, and similar to evidence described above with CIRT, there is suggestions of a dependence on HR in response to BNCT. A study performed in a single hepatocellular carcinoma cell model showed a time-dependent increase in BRCA1 and Rad51 protein levels following BNCT treatment, whilst there was no difference in the levels of Ku70/80 [41]. Similarly, an increase in Rad51/Rad54 mRNA expression, but not of Ku70 mRNA, in thyroid follicular cancer cells was observed compared with no changes in any of these genes following γ-irradiation [42]. The combination of these findings suggests a reliance on HR following BNCT that is potentially not tumour type specific. Conflicting data has been seen in CHO cells and mouse embryonic fibroblasts, highlighting the necessity of NHEJ for the repair of BNCT-induced damage, due to increased sensitivity of cells lacking Ku70/80 or Lig IV [43,44]. Although this data seems contradictory, it could be possible that rodent and mammalian cells rely on different repair pathways following BNCT treatment. Although not a specific focus of this review, it is also important to consider the cellular responses to other forms of high-LET radiation, such as other heavy ions and α-particles. Overall, the consensus is a significant increased dependency on HR following these types of radiation modalities [40,45–48] (some data summarised in Table 1). For example, utilising iron ion irradiations (1 GeV; 151 keV/µm), a dependency on HR has been shown due to increased sensitivity of both rodent and mammalian cells lacking Rad51 [45]. This phenotype was complemented by an increase in resistance of mammalian cells following S phase synchronisation where HR is most active, compared with cells in G_1_ that rely on NHEJ. Similarly, other studies focusing on heavy ions, such as iron, nitrogen and titanium with higher LET, highlight increased DNA end resection and further support the essentiality of HR in the repair of high-LET induced CDD [40,46]. Finally, studies performed using α-particle irradiation have drawn the same conclusions of an increased reliance on HR following this high-LET radiation modality [47,48].

Overall, there is an indication that CDD induced by high-LET radiation has an increasing dependence on the HR pathway for repair. However interestingly, there is contrasting evidence following CIRT, where there is a suggestion that NHEJ plays a more prominent role, and similarly lower LET PBT data suggest the involvement of multiple pathways (NHEJ, HR and BER) for the repair of the DNA damage induced. Consequently, a view that LET is the only determining factor in repair pathway dependence following IR is overly simplistic. There is various evidence to suggest that there is a plethora of contributing factors, which in different scenarios may produce a different outcome to the same form of radiation. One confounding factor is the type and distribution of the DNA damage induced, which will be somewhat related to the LET of the radiation, however, this can also be influenced by the chromatin state at the time of radiation and the cell cycle phase [49]. If the DNA breaks are generally uniformly distributed across the DNA and result in large, blunt fragments of DNA, such as that from low-LET IR, this will allow for binding of the Ku70/80 and therefore promote c-NHEJ. However, an increase in DSB density resulting in smaller fragments of DNA will prevent the binding of Ku70/80 [50] and therefore require end resection, which will ultimately favour a-NHEJ or HR. Furthermore, if euchromatin is exposed to IR, due to the occurrence of DNA replication and transcription, there will also be an increase in the requirement for end-resection and the final choice between a-NHEJ and HR would ultimately be a result of the available and activated repair proteins. If PARP-1 is bound to the exposed DNA, this would then favour a-NHEJ, however, if the DNA was coated with RPA this would result in HR. The cell cycle is another confounding factor in the damage following IR, not only affecting the susceptibility of the DNA to damage and the possible activation of the HR pathway, but this can also influence the activation and availability of repair proteins, due to cyclin-dependent kinase-dependent phosphorylation of these proteins [51]. Additionally, factors such as the cell and tumour type, dose and specific radiation source, will also be important contributors to repair pathway selection. Some of these factors have been more extensively reviewed [52].

## Therapeutic optimisation with high-LET radiation

Given that DNA is the major target for driving the therapeutic response to IR, the proteins involved in the DNA repair pathways can be specifically targeted to impede repair and therefore increase the RBE of the radiotherapy. However as discussed in the previous section, and due to the somewhat conflicting evidence on the specific DNA repair pathways employed following the different radiation modalities, it is difficult to specify a particular drug target as a focus in combination with a specific IR modality. Nevertheless, evidence is available for the impact of targeting key DSB repair proteins, such as ATM, ATR, DNA-PKcs and PARP-1 (summarised in Table 2). PARP inhibitors are thought to be particularly attractive in combination with low-LET radiation, by blocking the repair of the majority of DNA damage generated in the form of DNA base damage and SSBs, leading to the formation of more toxic DSBs during DNA replication. Indeed, extensive work has been carried out to examine the radiosensitisation of cells with PARP inhibitors following X-rays and other low-LET radiation, however comparative studies with high-LET radiation modalities are scarce. One study has characterised the comparative effect of olaparib in pancreatic cancer cells (MIA PaCa-2) following low-LET γ-rays and CIRT both at 13 and 70 keV/µm. This revealed an increased radiosensitivity with all three radiation conditions but particularly with the higher-LET carbon ion irradiations [53]. Radiosensitivity was associated with increased persistence in the levels of DSBs as analysed by γH2AX foci. Another study found that the PARP inhibitor talazoparib was able to reduce proliferation of glioblastoma (R633 and TG1) cells following low-LET photon irradiation, although these effects were exacerbated with high-LET CIRT (50 keV/µm), with particular effects on glioblastoma stem cells [54]. Similarly, in our study we previously have seen that olaparib specifically radiosensitised HeLa cells to relatively high-LET PBT (11 MeV; 12 keV/µm), due to the persistence of non-DSB CDD, as seen via an enzyme modified comet assay [55]. PARP inhibitors are well known to produce synthetic lethality in HR-deficient cells, including those with BRCA1/2 mutations, and this effect has also been observed in combination with IR. Indeed, olaparib has been shown to induce radiosensitivity of chondrosarcoma (CH2879) cells to photons, PBT (62 MeV; 11 keV/µm) and CIRT (95 MeV; 75 keV/µm), and genetic profiling determined that this could be a result of mutations in HR genes, such as Rad50 [56]. Supporting this, a study has combined the use of olaparib and B02 (a potent Rad51 inhibitor), to show radiosensitisation of non-small cell lung cancer (A549) and pancreatic cancer (KP4 and PANC1) cells to both low-LET photons and relatively high LET protons (1.3 keV; 25 keV/µm) [57].

**Table 2:** Comparative studies investigating effects of radiation LET on inhibiting various repair proteins

Target protein(s)	Compound(s)	Low-LET radiation	High-LET radiation	Cell type	Most effective IR modality	Ref.
**PARP-1**	Olaparib (AZD2281)	γ-rays	CIRT; 290MeV, Entrance Plateau 13kev/µm, Bragg peak 70kev/µm	MIA PaCa-2	CIRT; 70 keV/μm	[53]
**PARP-1**	Temozolomide (T2577), Talazoparib (BMN673), Olaparib (AZD2281), AG14361	X-rays; 225 kV	CIRT; 50keV/µm	R633, TG1	CIRT; 50keV/μm	[54]
**PARP-1**	Olaparib	X-rays; 100 kV	PBT; 58 MeV, Entrance Plateau 1 keV/µm, Distal-SOBP 12 keV/µm	HeLa	PBT; 12 keV/μm	[55]
**PARP-1**	Olaparib (AZD2281)	X-rays; 225 kV	PBT; 62 MeV, 11 keV/µm, Mid-SOBP CIRT; 95 MeV, 73 keV/µm	CH2879	Similar effectiveness	[56]
**PARP-1 and Rad51**	Olaparib (AZD2281), B02	X-rays; 225 kV	PBT; 1.3 MeV, 25 keV/µm, SOBP	A549	Similar effectiveness	[57]
**DNA PKcs**	NU7026	X-rays; 200 kV	CIRT; 290 MeV, 50 keV/µm, Mid-SOBP	H1299, A172, U251MG, MEF	Similar effectiveness	[58]
**DNA PKcs**	NU7026	X-rays; 6 MV	CIRT; 300 MeV, 49 keV/µm	MRC-5, A549	CIRT; 49 keV/μm	[59]
**DNA PKcs** **ATM**	M3814 AZD1390	X-rays	α-particles	H460, 22Rv1, HCT116, H1299, MRC-5, HEK293	X-Rays (DNA PKcs) α-particles (ATM)	[48]
**DNA PKcs** **ATM** **ATR**	NU7441 KU55933 VE-821	X-rays; 6 MV	PBT; 100 MeV, 9.9 keV/µm	H460, H1299, PANC-1, Panc 10.05	PBT; 9.9 keV/µm	[60]
**ATM**	AZD0156	X-rays; 6 MV	PBT; 76.8MeV, Entrance Plateau 2.2 keV/µm, Bragg Peak 7 keV/µm	BT549, U2OS, MDA-MB-436, V79, VC8	PBT; 7 keV/μm	[61]
**ATR**	VE-821	X-rays; 70-90 keV, 1-2 keV/µm	Iron ions; 1GeV, 150 keV/µm, α-particles; 5.49 MeV, 124 keV/µm	A549, HCT116	α-particles	[62]
**ATR**	VE-821	X-rays; 200 kV	PBT (SOBP); 124.7 MeV, 2.9 keV/µm, CIRT (SOBP); 238.6 MeV, 55.2 kev/µm	SW-1353, Cal78	PBT; 2.9 keV/µm, CIRT; 55.2 keV/µm	[63]
**ATR**	VE-821	X-rays; 200 kV	CIRT; 290 MeV, 70 kev/µm	HeLa, U2OS, 1BR-hTERT	Similar effectiveness	[64]
**CHK1**	PF-477736	X-rays	PBT; 230MeV, SOBP	MDA-MB-231/453, Hs578T	PBT	[65]
**CHK1**	AZD7762	X-rays; 225 kV	CIRT; 80.55 MeV/µ, 50 keV/µm	A459, H1299	CIRT; 50 keV/µm	[66]
**WEE1**	Adavosertib (MK-1775)	X-rays; 200 kV	CIRT; 290 MeV, 50 keV/µm, Mid-SOBP	H1299	Similar effectiveness	[67]

In terms of targeting DSB repair, it has been shown that DNA-PKcs inhibition (NU7026) in combination with X-ray irradiation or CIRT (50 keV/µm) in non-small cell lung cancer (H1299) cells was more effective compared with inhibiting the HR pathway (using the Rad51 inhibitor, B02) [58]. Interestingly, inhibition of HR had a reduced sensitising effect following CIRT compared with photons, suggesting that targeting DNA-PKcs involved in the c-NHEJ pathway is the most effective strategy to both high and low-LET radiation. Similar conclusions were drawn following DNA-PKcs inhibition (M3814) in a number of cancer cell lines that showed a sensitisation effect when combined with either low-LET photons or high-LET α-particle irradiation, but where the combinatorial response was more dramatic with photon irradiation [48]. In contrast, there is evidence that combining the DNA-PKcs inhibitor (NU7026) with CIRT (49 keV/µm) increases radiosensitivity to a greater extent than low-LET photon irradiation in a lung cancer (A549) cell model, mediated through significant delays in DSB repair [59]. Despite this, the evidence in these studies is difficult to compare and draw definitive conclusions due to differences in experimental set-ups, cell types and inhibitors used. Individual tumour cell variability in responses are supported by a study which explored the effects of various different DNA damage response inhibitors in combination with either photons or PBT (9.9 keV/µm) in non-small cell lung (H460, H1299) and pancreatic (PANC-1, Panc 10.05) cancer cells [60]. This study showed an increased RBE following DNA PKcs (NU7441) or ATM (KU55933) inhibition in combination with PBT. However, the addition of the ATR inhibitor (VE-821) only increased the RBE for the H460 cell line following PBT, compared with photons. Specifically focussing on ATM and ATR as targets for radiosensitisation, a study has investigated the effects of inhibiting these enzymes following photons and protons, particularly the effect of proton LET through irradiating U2OS and BT549 cells at the entrance plateau (2.2 keV/µm) versus at the Bragg peak with a higher LET (7 keV/µm) [61]. This revealed similar radiosensitising effects following ATR inhibition (AZD6748) across all irradiation conditions, however, inhibition of ATM (AZD1390) was shown to have an exacerbated effect specifically following relatively high-LET protons. The effectiveness of ATM inhibition in combination with protons at the Bragg peak versus those generated at the entrance dose was also demonstrated *in vivo* using xenograft models, which displayed an enhanced response in a HR-deficient model. Interestingly, ATM inhibition was observed to be less effective in reducing cellular survival in combination with α-particle irradiation in a variety of cancer cells compared with the irradiation in the presence of DNA-PKcs inhibition [48]. However, this study did show an increase in micronuclei formation following α-particle irradiation combined with ATM inhibition. Furthermore, a study in non-small cell lung cancer and colorectal cancer cells (A459, HCT116) revealed there is a shift in the dominant enzymes responsible for the G_2_/M checkpoint arrest following IR of differing LET [62]. Following photon irradiation, it was revealed that both ATM and ATR are responsible for the profound G_2_/M checkpoint arrest, however, in response to high-LET α-particles (124 keV/µm) there was an observed increased dependence on ATR. Therefore, this study suggested that targeting ATR should be more effective with high-LET IR. This is supported by a study comparing the radiosensitisation of chondrosarcoma cells (SW-1353, Cal78) to either photons or CIRT (55.2 keV/µm) in combination with ATR inhibition (VE-821) [63]. This revealed an increased radiosensitivity in these cells following high-LET CIRT, which was specifically mediated by a down regulation of HR proteins at both the gene and protein expression levels. In contrast with this, data suggest that inhibition of ATR (VE-821) can radiosensitise both U2OS and HeLa cells exposed to photons or CIRT (70 keV/µm) [64]. Ultimately therefore, there is no clear consensus on the most effective radiosensitisation strategy in combination with high-LET radiation, and more systematic and comparative studies are required.

In addition to targeting DSB repair, it is considered that inhibition of the cell cycle is a viable approach for increasing the therapeutic efficacy of IR. This is thought to be particularly effective in tumours with p53 mutations through the inhibition of checkpoint kinases CHK1 and WEE1, which are essential for G_2_/M checkpoint arrest. However, again the literature examining this combinatorial strategy with high-LET radiation is lacking. A study in triple-negative breast cancer cells revealed that the G_2_/M checkpoint arrest was more readily induced following PBT (230 MeV) compared with X-ray irradiation, and subsequently an enhanced radiosensitising effect of a CHK1 inhibitor (PF477736) following PBT was observed [65]. However, this effect is likely proton specific, rather than being related to LET given the low-LET nature of the PBT used. Nevertheless, a study in non-small cell lung cancer cells (A459, H1299) showed that although the CHK1 inhibitor (AZD7762) radiosensitised the cells to photon irradiation, these effects were exacerbated following high-LET CIRT (50 keV/µm) through a more profound abrogation of the G_2_/M checkpoint arrest and persistent DSBs [66]. This study therefore suggested that the effects of cell cycle inhibition could be more effective following high-LET IR. In contrast with this, a study targeting the WEE1 kinase (MK-1775) in non-small cell lung cancer cells (H1299) exposed to photons or CIRT (50 keV/ µm) revealed no significant difference in the degree of radiosensitivity, therefore suggesting no relation to LET [67]. Nevertheless, substantially more studies using PBT and high-LET particle ions are required to further understand the therapeutic potential of targeting cell cycle checkpoint proteins to increase the therapeutic efficacy of the radiotherapy modalities and identify any relationship to LET.

## Perspectives

**Figure 3:**
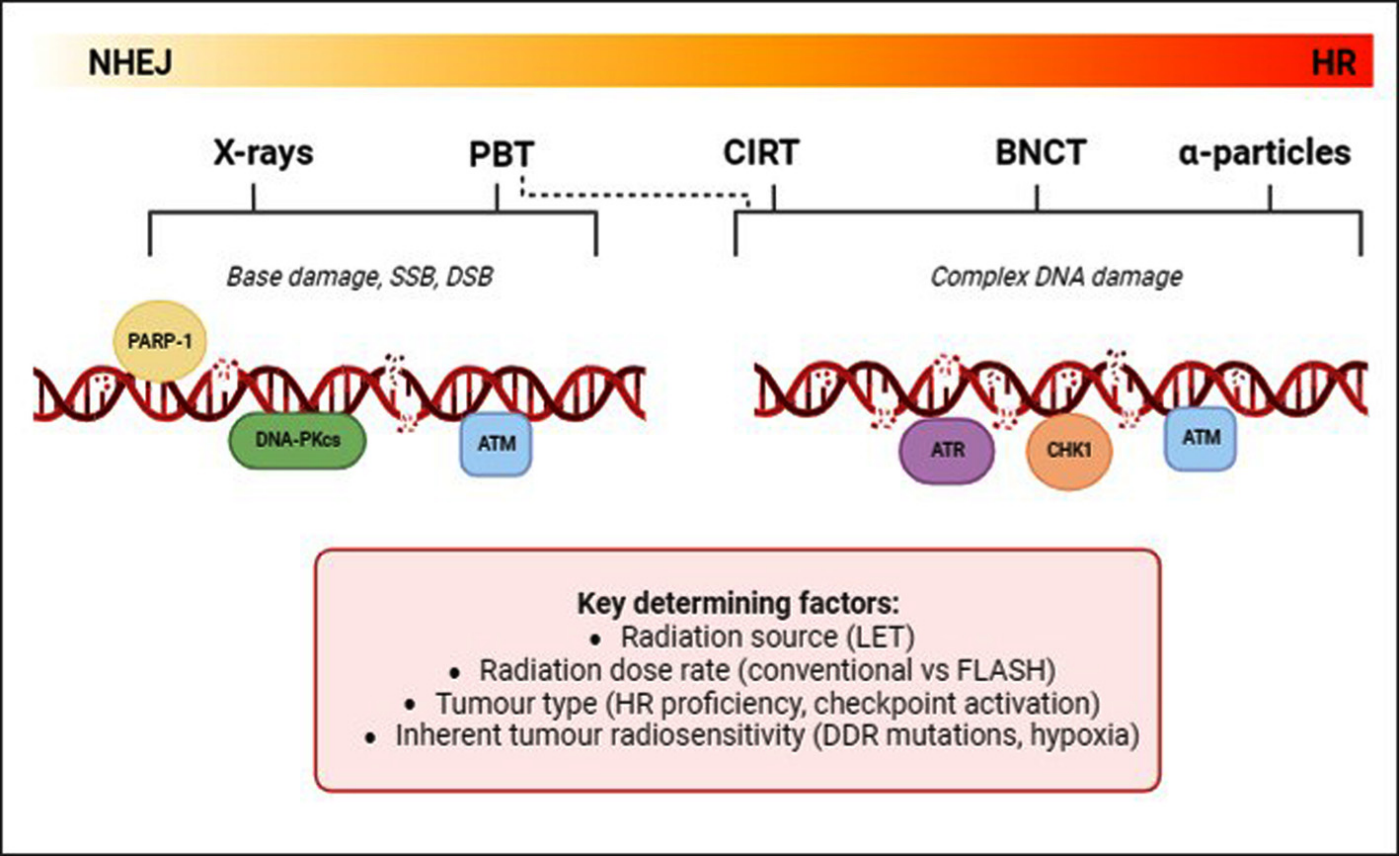
DNA repair protein and pathway dependence related to the LET of the radiation source X-rays and low-LET PBT result largely in base damage and SSBs which are repaired by the BER/SSB repair pathway, whereas DSBs which are less in frequency and simple in nature, rely mainly on NHEJ for repair. CDD is comparatively induced by high-LET irradiation (relatively high-LET PBT, CIRT, BNCT and α-particles) and evidence suggests an increased dependence on HR. Consequently, using inhibitors against PARP-1 and DNA-PKcs is thought to be more effective in combination with low-LET IR, whereas ATR and CHK1 may have more of an effect with high-LET IR. However, there are many factors affecting radiosensitisation and the dependence on specific DNA repair pathways, which include LET, dose rate, tumour type and inherent tumour radiosensitivity.

There is an increasing need for improvements in our biological understanding and the effective clinical utilisation of IR, due to the significant numbers of cancer patients (∼50%) receiving radiotherapy treatment. However, there are still significant uncertainties in the radiobiological effects inflicted by the various radiation modalities and also the individual tumour cell responses. These uncertainties include the varying effects across the different positions of the PBT Bragg peak, and also the impact of high-LET ions delivered by CIRT and BNCT. Despite this, high-LET radiotherapy is clearly the most promising with regards to optimal tumour cell killing effects, evidenced by the enhanced RBE generated through increases in the frequency and complexity of CDD that remains difficult for the cell to repair. However, it is not yet fully understood which specific DNA repair pathways are responsive to radiation of increasing LET, due to evidence suggesting an increased requirement for both HR and NHEJ in human cancer cells following PBT and CIRT, whereas there appears to be an increased reliance on HR following BNCT, other heavy ions and α-particle IR (Figure 3). The variability in this evidence could reflect the cell/tumour model being used, the specific radiation source, the LET, as well as the radiation dose and dose rate. These largely physical properties will have a significant impact on the damage inflicted and the resultant DNA repair pathways, although there are also biological factors to consider, including chromatin state, type and spectrum of DNA damage, NHEJ and HR proficiency, cell cycle stage, plus levels and possible mutations in key DNA repair proteins. Therefore, the cellular responses when examining different radiation modalities may not be entirely LET dependent. Furthermore, although it is beyond the scope of this review, there are also many immunomodulatory factors to consider that could influence the different tumour responses to IR of differing LET (reviewed in [68]). It is clear that more systematic studies need to be performed comparing well characterised cell lines with different radiation sources of increasing LET relative to clear biological endpoints (e.g. clonogenic survival, DSB and CDD levels, efficiency of BER, NHEJ and HR), but which is logistically challenging.

The utilisation of DNA repair, and to some extent cell cycle checkpoint, enzyme inhibitors as radiosensitizers has been widely proven with conventional X-rays in specific tumour types. However, this strategy in combination with PBT, as well as higher LET CIRT and BNCT has not been investigated in sufficient detail. This requires more investigative work comparing these various radiation modalities in combination with inhibitors such as those targeting PARP, ATM, ATR, DNA-PKcs and CHK1, to confidently determine a specific strategy that is efficacious in radiosensitising specific tumours. Again, it is clear that well characterised and specific tumour cell models are needed for this, and particularly given evidence that DNA repair capacity and HR proficiency of cells is one of the major determinants of the effectiveness of combinatorial treatment with IR. These studies should focus on identifying a clear relationship between radiosensitisation related to radiation type, with a particular focus on LET, through effects on CDD and repair. As well as data being generated in cell-based models, these should also be extended to 3D models (spheroids and patient-derived organoids) as well as *in vivo*, utilising mouse models, to further explore the translational potential of specific drugs in combination with certain types of radiotherapy. This would then enable the development of more targeted and specific treatment strategies resulting in improved clinical outcomes, with significant patient benefits.

## Abbreviations

ATM: ataxia telangiectasia mutated
ATR: ataxia telangiectasia and Rad3 related
BER: base excision repair
BNCT: boron neutron capture therapy
CDD: clustered/complex DNA damage
CHK1: checkpoint kinase 1
CIRT: carbon ion radiotherapy
DNA-PKcs: DNA-dependent protein kinase catalytic subunit
DSB: DNA double strand break
HR: homologous recombination
IR: ionising radiation
LET: linear energy transfer
NHEJ: non-homologous end-joining
PARP1: poly(ADP-ribose) polymerase-1
PBT: proton beam therapy
RBE: relative biological effectiveness
SOBP: spread-out Bragg peak
SSB: DNA single strand break
